# Seeing Neurodegeneration in a New Light Using Genetically Encoded Fluorescent Biosensors and iPSCs

**DOI:** 10.3390/ijms24021766

**Published:** 2023-01-16

**Authors:** David Stellon, Jana Talbot, Alex W. Hewitt, Anna E. King, Anthony L. Cook

**Affiliations:** 1 Wicking Dementia Research and Education Centre, University of Tasmania, Hobart, TAS 7000, Australia; 2 Menzies Institute for Medical Research, University of Tasmania, Hobart, TAS 7000, Australia

**Keywords:** neurodegeneration, dementia, high-throughput screens, biosensors, gene-environment interaction

## Abstract

Neurodegenerative diseases present a progressive loss of neuronal structure and function, leading to cell death and irrecoverable brain atrophy. Most have disease-modifying therapies, in part because the mechanisms of neurodegeneration are yet to be defined, preventing the development of targeted therapies. To overcome this, there is a need for tools that enable a quantitative assessment of how cellular mechanisms and diverse environmental conditions contribute to disease. One such tool is genetically encodable fluorescent biosensors (GEFBs), engineered constructs encoding proteins with novel functions capable of sensing spatiotemporal changes in specific pathways, enzyme functions, or metabolite levels. GEFB technology therefore presents a plethora of unique sensing capabilities that, when coupled with induced pluripotent stem cells (iPSCs), present a powerful tool for exploring disease mechanisms and identifying novel therapeutics. In this review, we discuss different GEFBs relevant to neurodegenerative disease and how they can be used with iPSCs to illuminate unresolved questions about causes and risks for neurodegenerative disease.

## 1. Introduction

Neurodegenerative diseases present with heterogeneous clinical and pathological traits, affecting different neuronal subtypes, non-neuronal cell types such as astrocytes, and diverse anatomical regions. For instance, movement is affected in both amyotrophic lateral sclerosis (ALS) and spinal muscular atrophy (SMA); whilst motor neurons are primarily affected in each disease, each have their own unique pathological mechanisms and, subsequently, the age of onset, clinical profile, and regions affected differ. The complexity of the nervous system and cell types within it are reflected in the heterogeneity of mechanisms leading to disease, and this has hampered the identification of treatments to slow, reverse, or halt disease progression. Consequently, neurodegenerative diseases impart a growing socioeconomic burden. 

Neurodegeneration may affect individuals at every stage of life; for example, the prevalence and incidence of the two of the most common neurodegenerative diseases—Alzheimer’s disease (AD) and Parkinson’s disease (PD)—are associated with an advanced age, whereas SMA is usually diagnosed at infancy. Chemotherapy-induced peripheral neuropathy (CIPN) is also a prevalent neurological side effect of cancer survivors (affecting 30–40%), and may affect individuals at any age, albeit a recent study reported the mean age to be 60.9 years [1,2,3]. With ageing populations, many nations are expected to see a significant rise in age-associated neurodegenerative diseases, and filling the mechanistic gap between epidemiological evidence and disease will help to prevent this increase [4].

Research into the complexities of neurodegeneration is challenging due to varied aetiologies, the wide range of age at presentation, and the involvement of different central and peripheral nervous system cell types. Genetics is the key factor causing familial cases of neurodegeneration. For examples, SMA is one of the most frequent autosomal recessive diseases and most common genetic causes of childhood mortality [5], and Huntington’s disease (HD) is a autosomal dominant neurodegenerative disease primarily affecting adults [6]. Even though environmental factors may contribute to varied clinical phenotypes [7], it is understood that the disease is purely genetic in aetiology. However, many neurodegenerative diseases, and particularly many associated with older age, such as AD, ALS, and PD, are more complex in aetiology and are most commonly a result of a combination of different factors, although rarer, fully penetrant familial forms do occur. For these diseases, age is a key risk factor, but one which is challenging to model in a laboratory setting. However, other factors, such as common gene variants or exposure to neurotoxic agents, and the potential for interactions to occur between these, can also modify the risk of disease and are more amenable to testing in a laboratory. For example, zoonotic transmission leading to variant Creutzfeldt–Jakob disease is due to the dietary exposure of bovine spongiform encephalopathy agent, but most individuals are homozygous for methionine at codon 129 on *PRNP*, leading to a higher susceptibility of the disease. For other causes of neurodegeneration such as trauma, genetics may influence the recovery rather than contribute to the initial insult [8]. 

Despite these varied aetiologies, neurodegeneration is characterised by a loss of function and structure of neurons, and frequently involves glial cells. However, the mechanisms leading to these changes are diverse and, in many instances, remain unresolved. To address this, there is a need to develop our understanding of complex aetiologies via ascertaining the temporal relationship between cell changes and then testing if these are mechanistically sequential or occur in parallel from an otherwise common upstream trigger. Answering these questions requires cost-effective, quantitative approaches amenable to high-throughput data acquisition. Live-cell imaging offers a robust tool for monitoring cell behaviours and, compared to the ‘snapshot’ provided in endpoint assays, enables the continual quantitation of cell changes and reduces artefacts produced via cell fixation or immunocytochemistry. However, live-imaging studies often utilise dyes, stains, and indicators that are transient, with long-term exposure to exogenous dyes increasing the toxicity to the cell and negating their ability to noninvasively monitor cellular health [9]. Advances in the field of fluorescent protein (FP) engineering has revolutionised ways of monitoring complex cellular processes that allow for stable genetic expression, high specificity, and spatiotemporal reporting in live cells. Here, we discuss the potential of these genetically encoded fluorescent biosensors (GEFBs) when expressed in human induced pluripotent stem cell (iPSC)-derived cell types to provide novel insights into the various genetic and environmental risks associated with neurodegenerative disease, and as platforms for the identification and pre-clinical testing of new therapeutic strategies.

## 2. Genetically Encoded Fluorescent Biosensor Advantages

**Figure 1:**
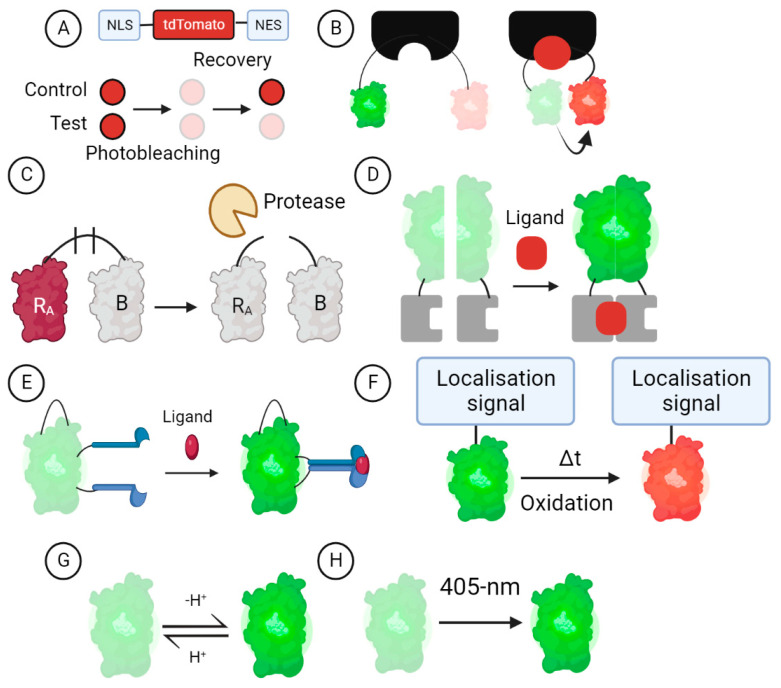
Eight different GEFB design concept examples. (**A**) Translocation of FP. (**B**) Fluorescence-resonance-energy-transfer-based biosensor. (**C**) Dimer-dependent GEFB. (**D**) Biomolecular fluorescence complementation. (**E**) A circularly permuted FP biosensor. (**F**) Oxidation-dependent fluorescent timer biosensor. (**G**) pH-sensitive FP. (**H**) Photo-transformable design. Each design is further elucidated in Section 3.1,Section 3.2,Section 3.3,Section 3.4,Section 3.5,Section 3.6 and Section 3.7.

Genetically encoded fluorescent biosensors are rationally designed chimeric FP-based molecular probes utilised for visualising cellular events [10]. There are varied GEFB designs (Figure 1) that have been reviewed extensively elsewhere [11]; in essence, GEFBs have a sensor that, for example, can be target sites of specific enzymes (e.g., caspase-3 proteolysis), causing an increase in the fluorescence of the reporting unit, which can be quantified in live cells over extended time periods. GEFBs may also have specific localisation tags that enable targeting to a desired cellular organelle such as the lysosome or mitochondria or for the quantitation of extracellular [12,13,14] and intracellular [15,16,17] analytes. Thus, GEFBs provide a quantitative measure of analyte levels, enzymatic activity, or specific pathway activity via high-throughput image acquisition and a subsequent analysis [18,19]. 

A key advantage of GEFBs is that they can be integrated into the genome of cell lines using CRISPR/Cas, notably the AAVS1 ‘safe harbor’ locus of iPSCs, with minimal off-target effects [20,21,22,23]. Such approaches enable the GEFB to be expressed in a ubiquitous manner in all derived cell types. Alternatively, GEFBs can be positioned under the control of cell-type-specific promoters, thereby restricting expression to certain cell types. Such an approach can be used in organoid models, where one wishes to determine the effects of a specific perturbation in neurons separately from astrocytes, for example. Another advantage of GEFB-based approaches is that they allow continuous data to be sourced from a single culture, thereby providing a temporal quantitation of cell changes from the same culture, ablating the need for multiple, cell-destructive end-point assays (Table 1). This is advantageous as it involves a high spatiotemporal resolution of cellular processes in specific culture mediums/treatment conditions, high data output, and less labour/time, and reduces the culture heterogeneity when compared to other methods that involve cell lysis or fixation, necessitating multiple cultures for collecting data from each timepoint. 

These advantages of GEFBs make them particularly well-suited tools for investigating pathological mechanisms in human cell-based models of neurodegenerative diseases. For example, iPSCs can be edited using CRISPR/Cas technology, allowing for the generation of disease-causing mutations and reversion to a consensus sequence to generate isogenic models of the genetic disease or to model the genetic risk of disease (e.g., *APOE4* and risk of AD) [32,33,34,35]. Furthermore, GEFBs would be powerful tools for evaluating the pre-clinical safety of emerging small molecular and genetic therapies before progressing to clinical trial [36,37,38]. 

## 3. GEFB Designs

GEFB technology offers innovative ways to measure various cellular interests; however, the development of novel GEFBs is time-intensive due to the need for rigorous testing and optimisation to ensure sensitivity and specificity for the targeted analyte [39,40]. The ease of use, kinetics, signal location, spectral overlap, and quantification need to be considered [41]. There is a range of GEFBs published in the literature and available from academic repositories (Table 2), including sensors for many processes and analytes relevant to neuroscience and neurodegenerative disease. Here, we briefly discuss various GEFBs designs most commonly used, which can be grouped into eight main categories [11], and highlight how they have been or could be useful to advance the knowledge of neurodegeneration. There are of course many additional GEFBs reported in the literature that enable the quantitation of other analytes and activities, and readers are pointed to reviews (e.g., [10,11]) that focus more broadly on GEFBs and that include a discussion of optimisation for many GEFB designs. 

### 3.1. Turnover and Translocation-Based GEFBs

The translocation of proteins from one compartment to another is central to cell homeostasis. One specific form of protein translocation, nucleocytoplasmic transport, is well-documented to be disrupted in some neurodegenerative diseases and involves the altered subcellular localisation of nuclear transcription factors and related proteins [56,57]. Nucleocytoplasmic transport activity can be quantified using the NLS-tdTomato-NES GEFB [43], which involves tdTomato fluorescent protein with an N-terminal nuclear localisation signal (NLS) and a C-terminal nuclear export signal (NES). This GEFB makes use of fluorescence recovery after photobleaching (FRAP), wherein a small region within a larger volume (i.e., cell nucleus) is illuminated for a short period at a high laser intensity, causing the photobleaching of NLS-tdTomato-NES, which is recovered through the diffusion of unbleached NLS-tdTomato-NES into the region of interest [58,59]. When nucleocytoplasmic transport is disrupted, there is a reduced nuclear localisation of unbleached NLS-tdTomato-NES (Figure 1A). NLS-tdTomato-NES has been useful for nucleocytoplasmic transport defects in models of ALS [43,60,61], deficient nuclear import in a model of HD [62], impaired nuclear import in a model of AD [63], and for showing that the global disruption of nucleocytoplasmic transport is not observed in models of spinal and bulbar muscular atrophy [64]. 

### 3.2. FRET-Based GEFBs

Fluorescence resonance energy transfer (FRET) GEFBs (Figure 1B) utilise two or more FPs that enable a shift in fluorescence from one fluorophore to another when the analyte of interest is bound to the sensor, producing a shift in the fluorescence emission spectrum that can be quantified [65,66,67]. Fluorescent indicator protein for thiamine (FLIPT) is a FRET-based GEFB consisting of thiamine binding protein sandwiched between cyan fluorescent protein and yellow fluorescent protein for the measurement of thiamine [45]. A recent study found a decreased protein content of *SLC19A3* (or thiamine transporter-2, ThTr2) and decreased thiamine in the cerebrospinal fluid (CSF) of HD patients [68]. Thiamine is an essential vitamin that plays a key role in maintaining brain function, including but not limited to: glucose metabolism, neuronal membrane conductance, and signal transmission, as well as nerve tissue repair [69]. Thiamine deficiency may be due to a multitude of reasons, such as malnutrition, gastrointestinal disorders, and chronic alcoholism [70,71,72], and leads to impaired energy metabolism due to mitochondrial dysfunction in focal regions of the brain, resulting in cerebral vulnerability [73]. High-dose biotin and thiamine treatment has been shown to ameliorate neuropathology in HD and biotin–thiamine-responsive basal ganglia disease [68,74]. FLIPT would prove to be a useful non-invasive real-time monitoring tool of thiamine in HD models. Moreover, FLIPT may be used to investigate thiamine deficiency’s role in astrocyte and synapse dysfunction [75,76,77] in neurodegeneration. 

### 3.3. Dimerisation-Dependent GEFBs

Dimerisation-dependent GEFBs (Figure 1C) involve a pair of non-fluorescing FP-derived monomers (copy A and B); copy A contains a quenched chromophore and copy B does not, although it serves to considerably increase the fluorescence of copy A upon AB heterodimer formation [78]. The AB heterodimer may be the starting point, fused together by a protease cleavable linker, with nonfluorescence therefore reporting the activity of a specific protease [79]. Dimerisation-dependent GEFB designs have previously been utilised for real-time monitoring applications of phosphoinositide (PI) signaling [80]. The diverse role of PIs in various processes such as signal transduction, membrane trafficking, and the regulation of the cytoskeleton make them important targets for furthering knowledge of their associations in disease, particularly the early-onset of AD associated with Down syndrome [81,82].

A second type of dimerisation-dependent GEFB uses biomolecular fluorescence complementation (BiFC), involving two non-fluorescent components that, when combined, fluoresce; however, unlike the design just discussed, each component is derived from a single FP (Figure 1D). As a result, two fragments from a single FP are each conjugated to proteins of interest and, when reconstituted, either via a protein/protein interaction or by an intermediary [83], are able to fluoresce. This design has been useful in generating BiFC GEFBs for detecting TAU aggregation [46] and alpha-synuclein cell-to-cell transmission [84]. In TAU-BiFC, full-length human TAU was fused to the N-terminal fragment of Venus and the C-terminal fragment of Venus. Under basal conditions, TAU-BiFC showed that the majority of TAU proteins exist as monomers, with cells exhibiting little fluorescence; however, chemically induced tau hyperphosphorylation increased the fluorescence intensity, therefore indicating higher TAU-TAU interactions. The alpha-synuclein cell-to-cell transmission GEFB also makes use of the Venus-based BiFC system. In this instance, however, one cell line stably expresses human alpha-synuclein conjugated to the Venus N-terminal fragment whereas another cell line stably expresses human alpha-synuclein conjugated to the Venus C-terminal fragment. In this way, the reconstitution of each fragment can only be visualised via the transmission of either fragment from one cell line to the other. It should, however, be noted that, when using GEFBs for studying protein–protein interactions, analysis should be accompanied by biochemical characterisation with complementary techniques [85]. 

### 3.4. cpFP GEFBs

The advent of circularly permuted (cp) FP has enabled the generation of another class of GEFBs, with the detection of analytes occurring as a result of the FP modification. In cpFP, the N- and C- termini are connected via a peptide linker that allows for a new terminus to be formed near the chromophore. In contrast to FP, cpFP has a diminished fluorescence intensity due to weak folding near the chromophore [11]; however, upon ligand binding, a conformational change in the sensory domain produces a measurable enhancement in the fluorescent intensity of the cpFP (Figure 1E) [86]. This concept has been extended using superfolder GFP (sfGFP), a modified GFP with an improved tolerance to circular permutation, increased thermodynamic stability, improved folding kinetics, and greater resistance to chemical denaturants [87,88]. In addition, through the introduction of chromophore-modifying mutations to change emission wavelengths, the GEFB could be altered to blue, cyan, and yellow for multi-colour imaging experiments [88,89]. Currently, this design has been successfully implemented in detecting calcium and multiple neurotransmitters, including glutamate, GABA, serotonin, dopamine, acetylcholine, and norepinephrine [47,49,50,51,52,53,90]. Neurotransmitter imbalances are common in neurodegeneration, notably the glutamatergic/GABAergic imbalance associated with excitotoxicity in AD, HD, and PD [91,92,93], as well as traumatic brain injury, a risk factor for neurodegeneration [94,95].

### 3.5. Oxidation-Dependent GEFBs

Another design strategy for GEFB is via a fluorescent timer [96]. For example, DsRed mutant (DsRed-E5) exhibits a green-to-red conversion over time following oxidation (dehydrogenation) of the Tyr-67 residue [54,97], thus proving useful for analysing time-dependent changes in the redox state (Figure 1F). A previous utilisation of this design includes MitoTimer [54], used to assess mitochondrial health. MitoTimer exhibits a GFP targeted to the mitochondria that shifts irreversibly to red upon oxidation. A significant shift towards red fluorescence, accompanied by an accumulation of red fluorescent puncta, signals mitochondrial stress and therefore would be useful in assessing mitochondrial dysfunction in neurodegeneration [98]. For example, MitoTimer has been used for investigating how excess alpha-synuclein affects mitochondrial homeostasis in PD [99].

### 3.6. Ion-Sensitive GEFBs

Ion-sensitive GEFBs are able to sense ionic changes (Figure 1G), including pH, using pH-sensitive FPs [30,55,100], a feature ideal for studying lysosome biology. Lysosomes are acidic organelles responsible for the degradation of both extracellular and intracellular macromolecules from endocytosis and autophagy, respectively [101]. Lysosomal storage disorders (LSDs) are characterised by lysosomal dysfunction and consist of over 70 diseases, most having a progressive neurodegenerative clinical course [102]. The lysosomal localised, pH-sensitive GEFB, RpH-LAMP1-3xFLAG, allows for the visualisation and measurement of intra-lysosomal pH, and, due to the presence of a FLAG tag, enables the isolation of lysosomes for subsequent analyses. Lysosomal acidification defects are implicated in the aetiology of LSDs and dementias [103,104,105,106] and are an important target for the treatment of these diseases [107]. pH-sensitive FPs are also useful for reporting events such as vesicle docking and fusion [108], and may be applicable to investigate the functions of acid-sensing ion channels that are widely expressed in the human brain, where its activation via acidosis may lead to neurodegeneration [109,110] by predisposing individuals to amyloid beta (Aβ) aggregation or inflammation [111]. These pH-sensitive GEFBs would therefore suit investigating differences in enzymatic activity or cellular processes associated with acidosis. 

### 3.7. Photo-Transformable GEFBs

Other FP properties, such as those in PA-GFP [112], mEos [113], or Dronpa [114], allow for GEFBs to be photoactivatable (Figure 1H), photoconvertible, or photoswitchable, respectively. These collective properties have been termed photo-transformable probes and are gaining traction for studying neuronal structure, connectivity, and function [115]. PA-GFP for example, has been used for neural tracing [116] and the measurement of protein diffusion across cellular compartments [117]. Attaching PA-GFP to a protein localisation signal for a particular organelle allows for the quantitation of intracellular protein trafficking between membranes [112,118]. Moreover, there is also a photoswitchable GEFB for the deep-tissue monitoring of cells, RpBphP1 [119], and photoactivatable Optopatch3, a genetically encoded voltage indicator [120].

## 4. Leveraging GEFB and iPSC Technologies for Pre-Clinical Applications

**Figure 2:**
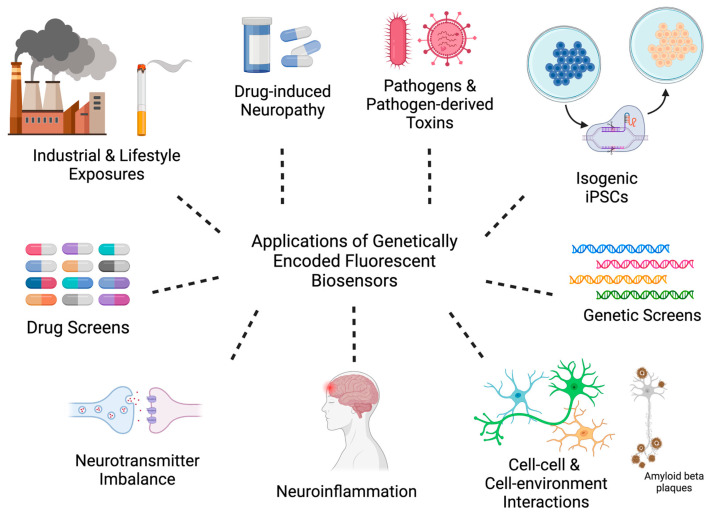
Overview of GEFB pre-clinical applications.

As described above, the aetiology of neurodegenerative diseases varies considerably, and there is much that remains unknown about how genetic and environmental factors, either singularly or in combination, prime cells for neurodegeneration. Whilst technologies such as CRISPR/Cas gene editing have made modelling genetic variants relatively straightforward [121,122], not all environmental factors will be able to be modelled using iPSC-derived cell types. However, there are many where such an approach is possible and is likely to provide new clues regarding neurodegenerative mechanisms. In this section, we will describe approaches to how GEFBs and iPSCs can be used in combination to better understand neurodegenerative mechanisms, test for the pre-clinical toxicity of novel therapies, and identify lead molecules in screening applications. It is important to note here that whilst we discuss the applications of GEFBs to pre-clinical research in discrete sections, there is significant potential for overlap in the experimental design, especially with regard to gene–environment interactions (Figure 2). 

### 4.1. Industrial and Lifestyle Exposures

One of the most immediate uses of GEFBs in iPSC-derived cell types is for investigating the effects of exogenous agents, and, in particular, the multitude of neurotoxic industrial and agricultural chemicals, antineoplastic and illicit drugs, and more widespread exposures, such as cigarette smoking, air pollution, or bushfire smoke [123]. Most of these agents are amenable to high-throughput applications, and thus quantifying the concentration and time of exposure effects of each using GEFBs on specific cell functions is relatively straightforward. Such an experiment would be expected to produce baseline data for use in subsequent experiments focused on mitigating the toxic effects. Other exposures, such as pathogens, are likely to require more sophisticated experimental designs to overcome, for example, the potential for bacterial growth when seeded into the iPSC-derived cultures.

#### 4.1.1. Pesticides

Although used as insecticides, herbicides, and fungicides, some pesticides present significant risks for neurodegenerative diseases in people. For example, chronic exposure to non-toxic levels of organophosphate pesticides such as tri-o-cresyl-phosphate, chlorpyrifos, and triphenyl phosphite are considered risk factors for ALS [124,125,126,127]. Indeed, people exposed to acute, higher levels of organophosphate pesticides acquire a condition called organophosphate-induced delayed neuropathy, which has many pathological similarities to ALS [128]. Other pesticides, such as paraquat and rotenone, are associated with PD [129]. 

Some agents have well-described modes of action. Rotenone, for example, inhibits the mitochondrial respiratory chain, leading to the production of reactive oxygen species via the release of inflammatory cytokines and autophagy inhibition [130,131,132]. However, the cellular consequences of many other pesticides have not been as extensively studied. A further understanding of how these pesticides affect brain cell types and elicit changes associated with neurodegenerative disease may yield new clues about disease mechanisms. Such studies could include quantifying the impact of specific pesticides on cell functions known to be disrupted in neurodegenerative disease, such as autophagy and mitochondrial health. 

iPSC-derived models of the blood–brain barrier (BBB) are also relevant to studying the contribution of pollutants to neurodegeneration. The BBB functions to prevent toxic substances entering the brain from the blood, filter harmful compounds from the brain to the blood, and supply the brain with nutrients [133]. Compromised BBB integrity may arise from pathogens, diesel exhaust inhalation, and diet via microglial activation and excitotoxic mechanisms that increase BBB permeability [134,135,136]. 

#### 4.1.2. Chemotherapy-Induced Peripheral Neuropathy

One of the most frequent side effects of antineoplastic drugs is CIPN, with the availability of treatments for this syndrome being very limited. This debilitating condition often presents in a ‘socks and gloves’ manner due to drug-induced neurotoxicity in the sensory neurons of dorsal root ganglia, such that the feet and hands are affected by pain and numbness [137,138,139]. Complicating our understanding of CIPN, and developing treatments for CIPN, is that different antineoplastic agents used for specific cancers have different mechanisms of action [140,141]. Although molecular mechanisms of CIPN are incompletely understood, common pathogenic mechanisms include altered calcium homeostasis and mitochondrial dysfunction [140], two parameters for which GEFBs have been characterised (Table 2). Thus, a toxicity screen testing different antineoplastic drugs on iPSC-derived neurons expressing these GEFBs could help to identify sensitivity thresholds for drug concentration or the duration of exposure. A subsequent molecular analysis of cells exposed to antineoplastic agents at the threshold may identify biomarkers of CIPN or reveal pathways critical to preserving the neuron structure or function. With regard to the latter, preventative treatments that are currently undergoing trial or have demonstrated a reduced frequency and intensity of CIPN include oral cannabidiol, lithium, and zinc [142,143,144], which could be pre-administered as part of combinatorial therapy.

#### 4.1.3. Pathogens and Pathogen-Derived Toxins

Multiple pathogens, including bacteria, viruses, fungi, and parasites, have been implicated in neurodegenerative diseases [145,146,147,148,149,150], ultimately contributing to disease pathology via neuroinflammation and other mechanisms. Recently, it has been shown that *Toxoplasma gondii* (*T. gondii*) has pathologically conserved mechanisms of infection in iPSC-derived glutamatergic neurons [151]. Infection with *T. gondii* is capable of causing signs of AD and has demonstrated an induction of Aβ immunoreactivity, hyperphosphorylated tau, elevated glutamate, autophagy, mitochondrial damage, and neuronal death [152,153,154,155]. The replication of these pathological mechanisms using GEFB expressed in iPSC-derived neural lineages would further elucidate the significant pathological mechanisms, especially as seroprevalence (the frequency of individuals in a population who test positive for *T. gondii* antibodies based on blood serum) is global and rates are reported anywhere from 0.5% to as high as 87.7% in some geographical regions [156,157,158,159,160].

Although less established as a risk factor than pesticides, exposure to cyanotoxins from cyanobacteria present in both aquatic and terrestrial ecosystems [161] may increase the risk for ALS [162], as well as other neurodegenerative diseases [163]. As with pesticides, cyanotoxin exposure causes a neurodegenerative condition in people that has features in common with ALS, including TDP-43 inclusions [163,164]. There are several molecules that may mediate these effects, including L-BMAA and its isomers 2,4-DAB and AEG [165], all of which are non-standard amino acids. L-BMAA is the most characterised of these and may act as an excitotoxin on AMPA/kainate receptors [166], but also may impart toxicity via incorporation into proteins [167,168]. How 2,4-DAB and AEG exert a neurotoxic effect, and if this differs from that of L-BMAA, is not yet established, but there is evidence that these are toxic to neural stem cells and that 2,4-DAB is the most potent neurotoxin [163]. Using iPSC-derived neural cells that express a range of GEFBs will add to our understanding of how cyanotoxins are neurotoxic and may provide insight into mechanisms of neurodegeneration. 

### 4.2. Genetics

Molecular genetics has advanced our understanding of aetiology and risk of developing neurodegenerative disease. Whilst advances in genetic technologies have accelerated identifying causal or predisposing risk variants, there remains a bottleneck associated with defining how each variant affects the biology of affected cell types. This is especially evident for ubiquitously expressed genes, such as those associated with multi-tissue diseases such as neuronal ceroid lipofuscinosis (Batten disease), where the brain and eyes can be affected [169]. 

#### 4.2.1. Isogenic Disease Models

The generation of human isogenic disease models allows for the in vitro culturing of two populations of cells that have a shared genetic background, but which differ at disease-associated loci. This provides a means to minimise the effect of gene variants inherent to distinct donors that may otherwise have a significant influence on disease-relevant cell phenotypes. Recent years have seen CRISPR/Cas technology become widely used for this purpose, and there are now many examples of isogenic models of neurodegenerative disease in the literature [170,171,172]. Large-scale projects combining CRISPR/Cas and iPSC technology, such as that of the Neurodegenerative Disease Initiative (iNDI), are producing dozens of isogenic cell lines modelling many variants associated with age-related neurodegenerative diseases [173,174]. 

Used in tandem with GEFBs, isogenic iPSC-derived cell types would enable unique comparisons between cells with or without pathological variants, offering a novel insight into disease progression. Such an approach may be particularly useful for gene mutations of unknown effects, such as private mutations seen in many families that cause rare and ultra-rare diseases or variants of uncertain significance (VUS), to ascertain whether they impact known targets to a greater or lesser extent than more prevalent variants. For example, in a Belgian AD cohort, a patient carried the VUS *PSEN1* p.P355S and *APP* p.G625_S628del, with limited knowledge of how these specific mutations in causal genes interact [175]. Such data are likely to be important for precision medicine applications, where knowledge of variant effects is needed to predict the safety and outcome of emerging therapeutic strategies. However, this approach relies on prior knowledge of which pathway(s) a specific disease-associated gene functions in and the availability of an appropriate GEFB, and therefore will not be a suitable approach for all gene variants associated with neurodegeneration. 

#### 4.2.2. CRISPR-Based Genetic Screens

Where gene function is not well understood, an alternative approach would be to screen the effects of gene perturbation in a targeted manner using iPSC-derived cell types expressing a specific GEFB. This concept has been used to investigate *C9ORF72* repeat expansion’s role in nucleocytoplasmic transport disruption using ALS patient iPSC-derived neurons; a reduced nuclear recovery of NLS-tdTomato-NES was observed in C9-ALS neurons compared to control lines [43]. Further expanding on this concept, researchers could investigate subtle or significant differences between different pathogenic alleles or gene–gene interactions that may additively or synergistically increase the disease risk. For example, rare variants of high-to-intermediate penetrance, in combination with common risk variants, may contribute to the genetic complexity of neurodegenerative diseases and lead to less/more severe disease phenotypes [176]. An area of particular interest would be furthering the knowledge of common variants and VUS that may interplay between different cell types, such as neurons and glia. Rare variants in microglia-expressed *TREM2* have been associated with an increased risk of AD by two to four-fold, whilst other variants reduce microglia-mediated neurodegeneration [177]. Understanding the genetic complexities driving pathogenicity across different brain cell populations will help to develop more informed combinatorial therapies.

### 4.3. Cell–Cell and Cell–Environment Interactions

For many neurodegenerative diseases, cell types other than neurons are involved in the pathological process and, in some instances, may be the primary trigger for the disease. For example, vanishing white matter disease is one of many astrocytopathies wherein astrocytes play a central role in driving brain pathology [178,179]. Relatedly, there is potential for the transmission of pathology from affected cells, as seen in prion diseases [180]. Thus, there are clearly cell-autonomous and non-cell-autonomous mechanisms of neurodegeneration that occur in specific disease contexts, which remains an important issue to resolve because it may be that different interventions are needed for diverse cell types. For example, CLN3 disease, one form of neuronal ceroid lipofuscinosis, is caused by variants in the ubiquitously expressed CLN3 gene [181]. CLN3 is expressed in neurons, astrocytes, and microglia, with each cell type exhibiting pathology [182,183,184]. Therefore, neurons in CLN3 disease may (i) be intrinsically vulnerable to CLN3 variants, (ii) have an altered ability to respond to signals from glial cells, or (iii) be directly impacted by disease-promoting actions caused by CLN3 mutation effects in glial cells. It may also be that these scenarios combine in a yet undefined manner to promote neurodegeneration. 

Here, we will highlight how GEFBs expressed in iPSC-derived cell types could be used to explore non-cell autonomous contributions arising from astrocytes and microglia to cause the degeneration of neurons. There are, of course, other cell–cell interactions relevant to neurodegeneration, such as those acting between cell types of the blood–brain barrier, which is beyond the scope of this review, but we note that iPSC-derived cell types expressing GEFBs can provide valuable information in paradigms beyond glial–neuron interactions.

#### 4.3.1. Neuroinflammation

Astrocytes and microglia have neuroprotective functions through the provision of trophic factors and removal of waste products; however, each of these cell types may acquire a pro-degenerative phenotype that can drive neurodegeneration. These pro-degenerative ‘reactive’ astrocytes and microglia are found in post-mortem tissue of many neurodegenerative diseases [185,186,187,188]. Neuroinflammation is the production by reactive astrocytes and/or microglia of pro-inflammatory mediators such as reactive oxygen species (ROS), cytokines, chemokines, and secondary messengers, which have a detrimental effect on neuronal viability and a central role in the pathophysiology of neurodegenerative diseases [189,190]. 

The contribution of non-neuronal cells to neurodegeneration can be explored using GEFB-expressing iPSC-derived cell types. In particular, the co-culture of neuronal and non-neuronal cells provides an opportunity to examine activities of specific pathways occurring in specific cell types (see also the discussion of Challenges and Caveats with using GEFBs below). One example of where this could be applied is to resolve the pro-neurodegenerative mechanism of alpha-synuclein in PD. Alpha-synuclein can act as a pro-inflammatory stressor that upregulates protein kinase C delta (PKCδ) in microglia, leading to the activation of NFκB, subsequent neuroinflammation, and dopaminergic neurodegeneration [191,192]. However, it is debated whether endogenous alpha-synuclein expressed by microglia triggers glial pathogeneses and phagocytic exhaustion, leading to neurodegeneration, or if neuronally derived alpha-synuclein accumulation is sufficient to cause PD [193,194]. Using a variety of different GEFBs, including those available to monitor autophagy and mitochondria dysfunction that are known to be impaired in PD [194,195], it would be possible to assess how microglia co-cultured with a healthy control or with PD iPSC-derived neurons impact cell health. 

#### 4.3.2. Neurotransmitter Clearance, Hyperexcitability, and Excitotoxicity

Pathogenic events involving the dysregulation of neurotransmitters have been implicated in neurodegeneration. In PD, an in vivo study has shown that chronic unregulated cytosolic dopamine alone is enough to cause neurodegeneration [196]. Glutamate is an excitatory neurotransmitter implicated in the regulation of neurogenesis, synaptogenesis, memory, and neuronal plasticity [197,198,199,200]. This is especially true of the action of glutamate at synaptic NMDARs; however, glutamate activity at extrasynaptic NMDARs can lead to neurotoxicity and cell death [201,202,203]. Extrasynaptic NMDARs have been found to often be concentrated at points of contact with adjacent processes such as axons, axon terminals, and glia [204]. Similar to NMDARs, the overactivation of AMPA receptors induces excitotoxicity and is a target for future neuroprotective drugs [205]. Experimentally, R-iGluSnFR1, a red fluorescent indicator alternative to iGluSnFR, created via the replacement of cpEGFP with cpmApple [206], could be utilised in neurons exhibiting excitotoxic pathological features. In addition, a co-culture with iGluSnFR astrocytes and microglia would help to visualise loss of homeostatic function in glia [207], when exposed to various pathological hallmarks such as alpha-synuclein oligomers [208]. Glutamate dynamics monitored in real-time using fluorescent imaging could then be juxtaposed upon the targeted delivery of exosomes expressing beneficial microRNA, e.g., miR-124-3p and antioxidants such as catalase [209,210,211,212,213]. A further characterisation of this targeted therapy could also be performed using multielectrode array analysis. 

Microglia may also contribute to excitotoxicity: in sporadic ALS, it has been found that microglia release glutamate into the extracellular space in response to soluble iron accumulation in the spinal cord [185,214,215,216]. Intriguingly, a form of neurodegeneration with brain iron accumulation (NBIA)—pantothenate kinase-associated neurodegeneration (PKAN) [217]—has characteristic iron accumulation mainly in the globus pallidus. PKAN exhibits cognitive decline and dementia, which are are features of NBIA, with psychiatric and behavioural symptoms resembling frontotemporal dementia, and motor symptoms presenting a clinical mimicry of ALS [218,219]. Excess glutamate would overstimulate glutamate receptors on neurons, leading to the dysregulation of intracellular calcium homeostasis, aberrant organelle function, elevated nitric oxide and free radicals, and activation of proteases and kinases, as well as pro-death transcription factors [220]. Given the association between iron accumulation and microglial glutamate release, it would be interesting to investigate the role of other heavy metals that have been associated with dyshomeostasis and as environmental neurotoxicants in ALS [221,222]. 

#### 4.3.3. Amyloid and Amyloid Plaques

The accumulation of amyloid plaques is considered a hallmark of AD, with many recent clinical trials seeking to reduce the plaque load using immunotherapy [223,224]. However, that plaque removal may not improve clinical scores and that plaques are also observed in healthy individuals imply that a more detailed understanding of the cellular consequences of exposure to amyloid oligomers and plaques is needed [225]. Recent studies have sought to define how different forms of amyloid affect iPSC-derived neurons more clearly, revealing an increased vulnerability of cells carrying familial AD-causing gene variants [226]. Others have reported transcriptomic [227], proteomic [228], and electrophysiological [229] changes in iPSC-derived neurons when exposed to oligomeric amyloid. Similar experimental paradigms using iPSC-derived cell types expressing GEFBs will further refine what is known about how amyloid affects not only the neuron function but also other brain cell types, such as microglia, that are implicated in amyloid plaque clearance [230].

### 4.4. Development and Testing of Novel Therapeutics

Using iPSCs for neurodegenerative disease modelling allows researchers to model Mendelian and complex human genetic signatures, with derived cell types retaining disease-associated phenotypes. That this can be performed using standardised conditions with good reproducibility makes iPSCs well-suited for therapeutic screening using GEFBs to report disease-relevant cellular responses [231]. 

The real-time monitoring of various neural lineages and specific cellular compartments, with a single-cell resolution and avoidance of false-positives from a low drug bioavailability (in vivo experiments), gives several advantages for utilising GEFB for drug screening [232]. Cell seeding into multiwell tissue culture plates, drug-culture medium distribution, and automated fluorescent imaging are also highly flexible and scalable, allowing for the accelerated development of clinically relevant data from in vitro iPSC-derived cells that are patient-specific [233]. Demonstrable beneficial effects in vitro are a bottleneck that GEFB ameliorates via high-throughput screening so that a reduction in in vivo models is achieved. Fluorescent assays are also less time and labor-intensive than radiolabeling and immunochemical staining [232]. Moreover, in some GEFB drug screen applications where a high resolution is not needed, a fluorescence microplate reader can be utilised to ascertain EC50 or IC50 values. Various GEFB approaches have shown great utility in drug discovery [232], including the use of an automated high-throughput calcium imaging assay with FRET-based calcium indicator, Yellow Cameleon 3.6, to screen compounds capable of treating endoplasmic reticulum calcium homeostasis disruption linked to familial AD, mutant *PSEN1* [234]. 

Patient-specific iPSC-derived neuronal cells can be used for assessing cell-based traits associated with disease-related mutations. Previously, an open-source cloud-based image processing and analysis platform (CELLXPEDITE) was used to screen compounds that revert the multiparametric disease profile of ALS back to that of healthy motor neurons [235]. This is advantageous when compared to the unidimensional characterisation of other screening methods for diseases such as ALS [236], as it allows for a simultaneous measurement of multiple parameters that would be more reflective of a complex disease. The CELLXPEDITE platform is available for neuroscience applications wherein iPSC-derived neurons, fluorescent reporters, high-throughput live cell imaging data, and potential drugs are used to assess even subtle activity perturbations and identify therapeutics that revert these disease phenotypes to that of healthy cells. Used in conjunction with a calcium GEFB, the platform was able to compensate for imaging artefacts, execute photobleaching corrections, identify single neurons, and extract and de-noise calcium transients [235]. Such a platform paves the way for identifying novel therapeutic molecules for the treatment of genetically perturbed and multifaceted neurodegenerative diseases. 

## 5. Challenges and Caveats of Using GEFBs

The main challenges to the wide-spread characterisation and adoption of GEFB-based iPSC models are the development of novel GEFBs that increase the range of pathways and analyses that can be quantified, improvement of multiplexing abilities, and availability of deep learning tools for high-throughput screening. Of these, the multiplexing of GEFBs and image analysis tools are of relevance regardless of which GEFBs are used, and advancements in each of these areas have been made, broadening the applicability of GEFBs to neurodegenerative disease research.

**Figure 3:**
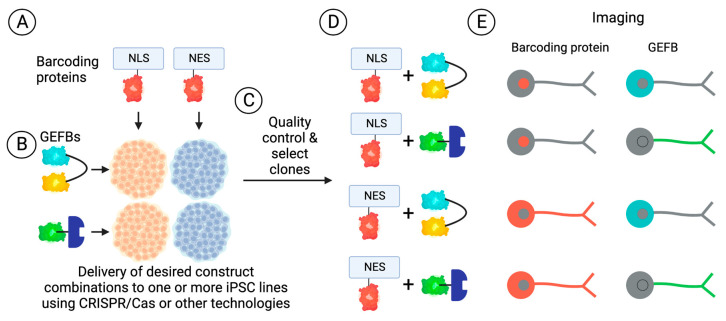
Overview of GEFB and barcoding protein knock-in to iPSCs for attaining multiplexed biosensor cell populations. iPSCs are modified to express barcoding proteins (**A**) or GEFBs (**B**) in pre-defined combinations, which following standard quality control steps for gene edited pluripotent stem cells (**C**), yielding cell populations for downstream experimentation (**D**). Imaging of barcoding proteins and GEFBs in iPSC-derived cell types can produce distinct patterns of fluorescence intensity and localization (**E**).

Multiplexing different GEFBs has been difficult due to the spectral overlap that can occur between different FPs used [80,237]. Such experiments are desirable because mapping whether certain processes happen in parallel or in series will refine our understanding of neuronal biology. Relatedly, identifying the temporal relationships between different perturbations may reveal if one or several interventions are needed to effectively halt pro-degenerative pathways. Past attempts to multiplex employed near-infrared GEFBs with common visible FP GEFBs and the generation of single fluorophore GEFB designs to avoid spectral overlap [238,239,240]. More recently, a method of ‘barcoding’ cells and using deep learning for image analysis was developed [241]. This involves labelling cells with different barcoding proteins, which ultimately consist of various red fluorophores attached to specific proteins with discrete subcellular localisations (e.g., nucleus versus cytoplasm; cell membrane versus cytoplasm; see Figure 3A). The barcoded proteins then enable a unique identification of cells, which would also express specific GEFBs (Figure 3B–D) with emission wavelengths between 450 nm and 550 nm (typically cyan-yellow-green fluorophore-based biosensors). Imaging of the resulting iPSCs or derivative cell types then enables a discrete identification of cells expressing barcoding proteins based upon the localisation of fluorescent signals to discrete compartments, which co-express particular GEFBs. For example, using two barcoding proteins and two GEFBs, four cell populations are potentially obtainable and able to be distinguished based on fluorescence intensities and localisations of the exogenous proteins (Figure 3E). For example, if placed under the control of appropriate promoters, barcoding proteins could be used to label specific neuronal cell types in genetically homogenous cultures or organoids. Alternatively, barcoding proteins could be used to label cells of a distinct genotype in pooled cultures, as used in larger-scale iPSC-based studies, where cell/donor identity is inferred using single-cell sequencing technologies [242,243,244], thereby increasing the throughput, decreasing the cost, and minimising potential artefacts from handling multiple cultures in parallel.

**Figure 4:**
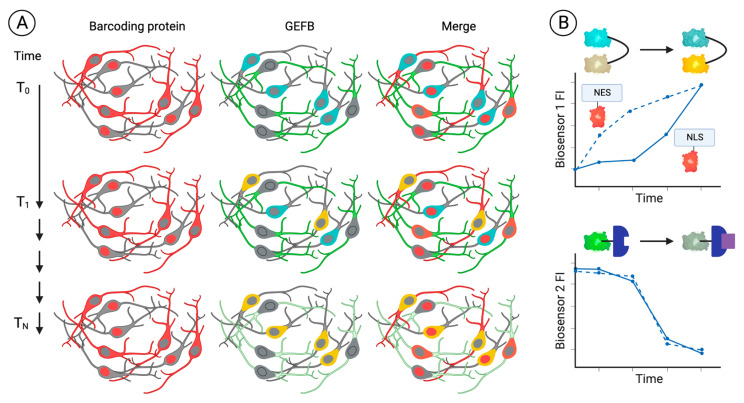
Barcoding proteins and GEFBs enable cell-specific quantitation of cell responses in multiplexed cultures. In pooled cultures of neurons expressing barcoding proteins and GEFBs, barcoding protein expression would remain stable over time, whereas GEFB expression changes (**A**). Quantitation of GEFB imaging can reveal changes occurring at different or the same rate in specific barcoded cell populations (**B**).

Barcoding proteins potentially allow for a live-cell recording of GEFB-based measures in different cell populations, utilizing, for example, pooled cultures of barcoded neurons, where cell identities are inferred via the unmixing of cells expressing discrete protein barcodes in images obtained using fluorescent microscopy (Figure 4A), enabling a quantitation of GEFB-based assays in each cell population (Figure 4B). Such models would enable the quantitation of changes occurring in cells of one genotype compared to another, or could, for example, assess how genotypes influence susceptibility to neurotoxins or other insults [245,246]. For example, using the four cell populations defined in Figure 3 and assuming that the localisation of red-fluorescent barcoding proteins represents the genotype, from one pooled culture, it is theoretically possible to determine that, over time, the genotype influences one GEFB responsive to a specific analyte or process, but not the other. Other approaches could include the perturbation of homeostasis in defined cell types, such as astrocytes that are co-cultured with neurons expressing GEFBs, or cultures where pooled cells of the same genotype are exposed to the same stimulus (for examples, see Figure 2) but express different GEFBs, thereby enabling the acquisition of multiple readouts from a single culture. 

In addition to the considerations relating to each specific GEFB, a major drawback of this approach is the number of discrete genetic modifications needed to generate libraries of barcoded iPSC lines that also express specific GEFBs; each cell line to be used requires two manipulations: one to barcode the cell line and one to deliver the GEFB. Clearly then, such models will only be developed with specific goals in mind, and will likely need to be developed for each specific application. A second limitation for GEFBs is that they rely on the expression of an exogenous protein, often of a considerable molecular weight, and potentially at a level that impacts negatively on cell homeostasis. Thus, for each GEFB, it is imperative to implement a considered approach that compares the engineered cells to those of the parental cell line, and that, where possible, employs positive control reagents to confirm similar responses between them. This may include comparisons of canonical cell functions, such as using electrophysiology for neuronal activity, glutamate clearance by astrocytes, and phagocytosis by microglia. For organelle-specific GEFBs such as those targeted to lysosomes or mitochondria, ensuring that organelle functions are not perturbed may also be appropriate. Other useful control experiments could employ small molecule activators/inhibitors of the pathway(s) being studied to ensure similar kinetics of response, such as staurosporine to induce apoptosis or rapamycin to induce autophagy.

A further caveat to studying neurodegeneration using GEFBs is that, for some applications, their use relies on a priori knowledge of which molecules, signaling events, and pathways are associated with the disease, and thus a limitation of this approach is that it is biased relative to techniques such as single-cell RNAseq. Relatedly, the choice of promoter used to control the expression of the GEFB may be a limitation in some experimental designs and should be an early consideration when planning gene-editing steps. However, the multitude of GEFBs available [10] could be used in various paradigms (see Figure 2 and related text) to reveal new associations of particular analytes or signaling events to specific neurodegenerative disease-associated genes or stressors, thereby seeding new research directions. 

## 6. Conclusions

The advent of GEFB technology allows us to quantify live-cell spatiotemporal data in a high-throughput manner, such that sequences of pathological events can be time course identified and made genetically relevant to any individual using patient-derived iPSC lines. As neurodegenerative diseases are complex multifactorial enigmas, this combination of GEFBs and iPSC technology will help to reveal an unseen world and unravel how each factor interplays with one another. Notwithstanding the limitation of complex and time-consuming novel GEFB generation, as global research efforts continue to develop and share designs, the arsenal of investigative tools will lead us to more effective therapies.

## Figures and Tables

**Table 1:** Comparative analysis of other methods to GEFBs.

Technique	Example(s)	Advantages	Disadvantages Compared to GEFBs	Reference
Specialised analytical devices	Seahorse XF HS Mini Analyzer (Agilent); Multi-electrode arrays	Allows for real-time data acquisition and continuous monitoring.	Usually, samples small areas near the sensor and measurements are indirect calculations. Does not allow for visualisation of cellular compartments.	[24,25]
Endpoint assay	Many cell-based assay kits from various companies	Quantitative measurements, high-throughput, with convenient and economic kits available.	Does not allow for continuous high spatiotemporal visualisation in living cells or tracking at single-cell resolution.	[26,27]
Organic dyes	Calcium indicators (e.g., FURA-2)	Quick to use; little preparation needed.	Relatively more invasive, long-exposure can lead to accumulated toxicity. Extended excitation can lead to more photobleaching. Short-term imaging. Dye leakage significantly contributes to accuracy. Difficulty monitoring activity in specific cell types and specific subcellular compartments.	[28,29]
Fluorescent pH probes	HPTS, SNARF-1, Lysotracker	High spatiotemporal resolution, long-term fluorescent and structural stability.	Difficulty in penetrating the cell membrane, and targeting methods to subcellular locations can perturb the cell and affect pH in the long run. May exhibit rapid photobleaching.	[30,31]

**Table 2:** Selected GEFBs relevant to neurodegenerative disease.

Design	GEFB	Sensing	FP	Reference	Addgene Plasmid Number
Turnover and translocation of FP	GFP-LC3-RFP-LC3ΔF	Autophagy	GFP and RFP	[42]	84572
Turnover and translocation of FP	NLS-tdTomato-NES	Nucleocytoplasmic transport defects	tdTomato	[43]	112579
FRET	LSSmOrange-DEVD-mKate2	Caspase 3	LSSmOrange and mKate2	[44]	37132
FRET	FLIPT	Thiamine	CFP and YFP	[45]	N.A. *
BiFC	Tau-BiFC	Tau–tau interaction	Venus	[46]	N.A. *
cpFP	GACh2.0	Acetylcholine	cpGFP	[47]	106073
cpFP	MatryoshCaMP6s	Calcium signalling	LSSmOrange and cpEGFP	[48]	100025
cpFP	GRAB_DA_	Dopamine	cpEGFP	[49]	113050 and 113049
cpFP	iGABASnFR	GABA	cpSFGFP	[50]	112176
cpFP	iGluSnFR	Glutamate	cpGFP	[51]	41732
cpFP	GRAB_NE1M_	Norepinephrine	cpEGFP	[52]	123309 and 123308
cpFP	iSeroSnFR	Serotonin	cpSFGFP	[53]	128484
Oxidation-dependent	MitoTimer	Mitochondrial health	GFP and DsRed1	[54]	52659
Ion-sensitive	RpH-LAMP1-3xFLAG	Lysosomal pH	pHlourin and mCherry	[55]	163018

* N.A. = not available from AddGene at time of writing.

## Data Availability

Not applicable.
